# Incorporation of Sea Spaghetti (*Himanthalia elongata*) in Low-Salt Beef Patties: Effect on Sensory Profile and Consumer Hedonic and Emotional Response

**DOI:** 10.3390/foods13081197

**Published:** 2024-04-15

**Authors:** Artur Głuchowski, Emily Crofton, Elena S. Inguglia, Maurice G. O’Sullivan, Joe P. Kerry, Ruth M. Hamill

**Affiliations:** 1 Food Quality and Sensory Science Department, Teagasc Food Research Centre, Ashtown, D15 KN3K Dublin, Ireland; emily.crofton@teagasc.ie (E.C.);; 2 Food Gastronomy and Food Hygiene Department, Institute of Human Nutrition Sciences, Warsaw University of Life Sciences (WULS), 02-776 Warsaw, Poland; 3 School of Food and Nutritional Sciences, University College Cork, T12 E138 Cork, Ireland; maurice.osullivan@ucc.ie (M.G.O.); joe.kerry@ucc.ie (J.P.K.)

**Keywords:** sensory, salt reduction, beef patties, sea spaghetti, *Himanthalia elongata*, seaweed, CATA

## Abstract

Seaweed is a naturally rich source of nutrients and exhibits techno-functional properties that are under study for their potential as ingredients in meat products. However, seaweed is associated with a particular flavor profile, and optimization of the sensory profile should be conducted alongside technical performance. This study investigated the feasibility of the application of sea spaghetti (*Himanthalia elongata*) in the production of low-salt beef patties and recorded the associated sensory profile and consumer hedonic-emotional response. Eight beef patty formulations with varying salt (0–1%) and seaweed (0–5%) contents were subjected to quantitative descriptive analysis via a trained sensory panel (*n* = 8) and six the formulations were selected for consumer testing (liking, emotional associations, saltiness perception, and purchase intent) by a group of 105 Irish resident consumers. The trained panel results showed that the intensity of seaweed odor, flavor, and visual presence in burgers was negatively related to the intensity of beef odor and flavor and that seaweed addition (5%) significantly increased the saltiness perception of low-salt burgers. Burgers with 1% added seaweed, although perceived by consumers as less salty, could substitute NaCl in low-salt beef patties without deterioration of their liking among regular burger consumers. Consumers associated all seaweed-containing samples, especially those containing 1% of sea spaghetti, with being good, pleasant, satisfied, and warm. The higher inclusion of sea spaghetti (2.5%) led to significantly lower overall liking and reduced purchase intent, while consumers associated this formulation with emotions such as being more adventurous, aggressive, and wild. Consumers who rejected seaweed burgers had the highest level of food neophobia and avoided foods with additives. The results demonstrate that 1% sea spaghetti seaweed can be successfully incorporated into low-salt beef patties, resulting in hedonic and emotional benefits without significantly increasing the salt content.

## 1. Introduction

European consumers are increasingly focusing on health and sustainability, leading to a rise in plant-based products and a clean-label trend [1,2,3]. In the Western world (Europe, America, and Oceania), consumers are becoming more focused on natural food manufacturing processes and ingredients, with some processes or ingredients being perceived as less natural and others as unhealthy [4]. Consumers increasingly want to be able to identify and understand the ingredients of food and beverages that they buy [3]. The lack of a clear definition of “clean” and the presence of numerous functional additives are posing new challenges for manufacturers and ingredient producers, particularly in the meat industry [5,6].

Beef burgers are a popular product in the Irish market, with 56.2% of consumers estimated to eat them at least once a month [7]. A survey by the Food Safety Authority of Ireland [8] revealed that burger samples from the Irish market have variable beef content and also include some food additives listed on the food label. While a beef patty is generally not considered a highly processed product, salt and fat reduction is usually possible, but when it comes to improving the health profile, such as reduction in fat or salt content, this can lead to associated compromises on sensory (flavor, juiciness, mouthfeel) or technological performance of the patties. Seaweed, fibers, and powdered vegetables are ingredients with functional properties that demonstrate high potential to be used in clean-label, healthier meat product formulations [6].

Marine macroalgae, popularly called seaweeds, have under-exploited potential as high-dietary-value ingredients in food products. Seaweed can be classified into three main taxonomic groups: *Rhodophyta* (red), *Phaeophyta* (brown), and *Chlorophyta* (green). They can be produced without a requirement for dedicated arable land, and it is possible to sustainably harvest certain seaweeds from shore or to farm them in coastal regions. Seaweed contains a wide range of valuable nutrients, such as protein, minerals, and fiber. As food ingredients, they also possess functional properties, whether through being flavoring agents to promote an umami taste or through providing texturizing properties, which has led to many culinary innovations [9,10]. Edible seaweed and/or their extracts have been added to various food products, including meat products (pork, beef, chicken, and seafood) or foods of plant origin (bread, pasta, and noodles), as thoroughly reviewed by Roohinejad et al. [11]. However, most of the meat-based studies have focused on pork [12,13,14,15,16], with little attention given to its application in beef products. Meat products with seaweed allow for maintaining the organoleptic and technological characteristics of products formulated with a lower salt content [17].

Due to the important role of salt in processed meat product attributes such as water-holding capacity, protein, and fat-binding, as well as maintaining product safety/shelf life and for sensory reasons, salt reduction is challenging [18]. Various dried seaweed species may contain 5.4–15.3% salt, with a generally low ratio of sodium to potassium (Na:K), as well as a significant content of amino acids, which contribute to their distinct umami taste [19].

The high content of minerals in seaweed material confers the possibility of using them as salt replacers in meat products, reducing the need for chemical salt and also potentially providing a source of other nutritious and flavorsome minerals [17]. Furthermore, the umami taste imparted by seaweed can play a significant role in NaCl reduction by providing similar flavor enhancement as salt [20]. The odor-induced umami enhancement from using natural ingredients such as seaweed can be used to create more palatable, clean-label foods [21]. Due to the characteristic flavor profile of seaweed, it is also possible that undesirable properties may be conferred on the product, for example, the appearance and aroma may be less accepted, especially by consumers unfamiliar with seaweed-based products [17,22]. For this reason, the inclusion quantities of individual seaweeds must be optimized. This is especially justifiable in the case of beef, in which consumers’ decision to buy beef is strongly related to its sensory attributes, and congruent eating quality is one of the key features [23]. Food neophobia, a growing phenomenon in countries where new products are not part of culinary tradition, negatively impacts consumer attitudes and reduces willingness to consume seaweed [24].

A growing body of literature has demonstrated the effect of the addition of algae or their derivatives to beef burgers [25,26,27,28,29,30,31]. However, there is still a significant knowledge gap regarding the properties and acceptability of the addition of dried sea spaghetti (*Himanthalia elongata*) to beef patties, especially in terms of sensory aspects. More studies are needed to understand how reformulation affects consumer acceptability of the final product, primarily focusing on sensory characteristics [29]. The aim of the research was thus to evaluate the potential application of sea spaghetti in the production of low-salt beef patties and its effect on the sensory profile and consumers’ hedonic and emotional responses. The specific objective of this study was to identify, describe, and compare consumer segments based on differences in individual food choices, i.e., reluctance toward novel foods. We also hypothesize that the addition of seaweed can enhance the saltiness perception in a low-salt version of a popular meat product, i.e., a burger patty.

## 2. Materials and Methods

### 2.1. Study Material

**Figure 1:**
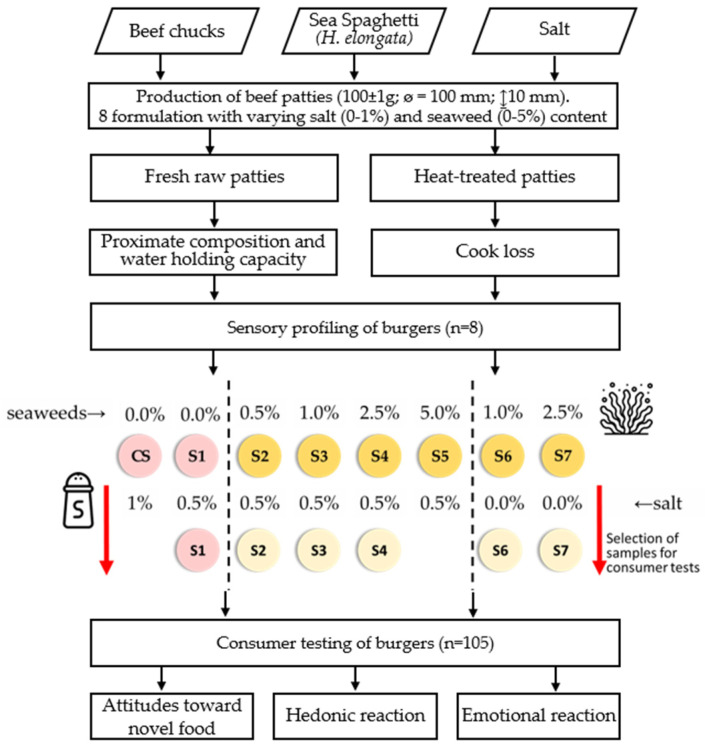
Study design on the effect of sea spaghetti (*Himanthalia elongata*) incorporation in low-salt beef patties on sensory characteristics.

The study involved the production of 8 formulations of beef patties with varying salt (0–1%) and seaweed (0–5%) contents and the establishment of sensory profiles, as well as consumer tests of 6 selected burgers (Figure 1). In consumer tests, only the most promising formulations were selected. A high content of salt could create biases among untrained panelists, especially in consumer salt perception.

All beef patties were prepared in the Meat Industry Development Unit of the Teagasc Food Research Centre (Dublin, Ireland). Beef chucks (Kepak Group, Clonee, Ireland) were diced and coarsely processed through a 7.5 mm plate in a mixer mincer (La Minerva Mixer Mincer, Bologna, Italy) and then manually mixed with table salt (Redbrook Ingredient Services, Dublin, Ireland) and dried sea spaghetti (*Himanthalia elongata*) procured from Wild Irish Seaweed (Caherush Point, Quilty, Co. Clare, Ireland) according to the study design (Figure 1, Table 1). The characteristics of the seaweed used in this study were fully described by Mohammed et al. [19]. After maceration for 15 min, the meat batch was further minced using a 3 mm mincer plate (La Minerva Model No. CIE701, Bologna, Italy). The samples of each formulation (100 ± 1 g) were placed between round wax paper and molded using a 10 mm hamburger patty press (Vevor, Cannock, UK), blast-frozen within 60 min, vacuum packed, and stored at −20 °C until sensory analysis. The fat content of samples was fixed for all formulations at 15%. Photographs of raw burger samples are available in Supplementary Materials (Figure S1).

### 2.2. Proximate Composition, Cooking Loss, and Water-Holding Capacity Analyses

Raw burgers were thawed overnight in the refrigerator before being homogenized in a Robot Coupe (R101, Robot-Coupe S.N.C., Vincennes, France). Moisture content was determined using the air drying method by AOAC (1991) procedure no. 950.46, and fat concentration was determined using the NMR Smart Trac Rapid Fat Analyzer (CEM Corporation, Matthews, NC, USA) by AOAC (1990) method no. 985.26. Protein content was assessed using an FP628 (LECO Corp., Benton Harbor, MI, USA) by AOAC (1990) method no. 992.15. Gravimetric weight loss on ashing the samples in a muffle furnace at 525 C (920.153, AOAC, 1990) was used to measure ash concentration. The Mohr method [32] was used to assess salt concentration by titrating chloride anions in ashed samples with silver nitrate. All of these analyses were repeated 4 times.

Samples were cooked in a water bath at 75 °C until they achieved an internal temperature of 72 °C. Cook loss was calculated as a % of raw patty weight. To measure water holding capacity, 5 g of raw beef patty was put on quantitative filter paper (Whatman number 1) and wrapped in cotton wool before being placed in a centrifuge tube. The tubes were then centrifuged for 10 min at room temperature at 4000 rpm. Following centrifugation, the cotton samples were removed and weighed. The water-holding capacity values were expressed as a percentage of the initial weight.

### 2.3. Sample Preparation and Presentation

Samples were thawed at refrigerated temperature (4 °C) for 24 h before the sensory analyses. Beef patties were grilled using a clamshell-type grill (Velox, Oxfordshire, UK). The process of two-sided heating at 170 °C was performed until 75 °C [33] in the burger patty core was reached (2.0 ± 0.5 min). The temperature was monitored using a calibrated thermocouple (TH103TC, Eurolec Instrumentation Ltd., Carrickmacross, Ireland). Beef patties were then cut into eights and two pieces (≈18 g) were either wrapped in aluminum foil (sensory profiling) or placed on a white paper plate (consumer testing). Samples were labeled with a 3-digit code and immediately presented in a sequential monadic order to assessors according to a randomized design. Between samples, the assessors were asked to cleanse their palates using filtrated water and plain crackers (Carr’s, London, UK) to minimize carry-over effects. Informed consent was given by all participants for their data to be used and analyzed.

### 2.4. Sensory Profiling

A trained sensory panel (n = 8; all female) evaluated the burger patties using Quantitative Descriptive Analysis (QDA) [34]. Sensory profiling was carried out according to the procedures of ISO 13299:2016 [35] and AMSA [36] guidelines under white light in a sensory laboratory (Teagasc, Dublin, Ireland) that meets the requirements of ISO 8589:2007 [37]. Sample evaluation data were collected using Compusense^®^ Cloud Software (Guelph, ON, Canada).

The trained sensory panel had extensive experience (>6 years) in the descriptive profiling of beef [38]. The panel participated in an additional three two-hour training sessions during which they identified and agreed to a list of 9 attributes that described the appearance, odor, texture, flavor, and taste of the burger samples. Panelists were trained and calibrated on each attribute through repeated exposure to a range of reference materials (Table 2).

Panelists evaluated the burgers in two repetitions throughout two 1 h sessions between 10 a.m. and 12.30 p.m. A 30-minute pause was implemented between sessions. A warm-up sample was presented at the start of each session to decrease first-order bias and calibrate the panelists. Panelists rated the samples following the procedure established during panel training. The intensity of sensory attributes was measured on a 10 cm unstructured linear line scale, with end-of-scale anchors ranging from none on the left to very strong on the right.

### 2.5. Consumer Testing

Consumer testing took place in the sensory suite in the School of Food and Nutritional Sciences at University College Cork during the morning. Participants were invited to the study from the UCC database using a convenience sampling method and were given clear instructions on how the experiment would proceed. Consumers provided written, informed consent and were offered a EUR 20 voucher to compensate for their time. Each consumer tested all 6 samples in a randomized order.

#### 2.5.1. Survey

The questionnaire (Supplementary Materials; File S1) was composed of three separate sections. The first section of the questionnaire covered formal consent for participation in the study. The second section investigated social-demographic characteristics (gender, age, education, financial situation, and residence place), beef burger consumption frequency and preferred consumption venue, and intensity of hunger sensation using a validated scale developed by Friedman et al. [39]. Consumers’ previous experience with seaweed and the level of their habitual dietary salt intake using a short validated questionnaire by D’Elia et al. [40] were also used. The salt habit assessment consisted of five questions with three responses and assigned scores ranging from 1 to 3. The actual total scores varied from 6 to 13 (possible range: 5–15), with higher values indicating a higher salt intake. Participants with 5–9 points were considered to have an average salt intake, whereas those with 10 or more points were considered to have a high salt intake [40].

Food neophobia level was computed using the Food Neophobia Scale (FNS) by Pliner and Hobden [41], attitudes towards seaweed were evaluated using questionnaire items proposed by Losada-Lopez et al. [24], and clean-label attitudes were evaluated using questionnaire items developed by Saba et al. [42]. All these statements were assessed on a 7-point scale, which ranged from 1—“strongly disagree” to 7—“strongly agree”.

The third section of the questionnaire was the sensory assessment. The degree of liking of aroma-, appearance-, flavor-, texture-, and overall liking were assessed by participants on a 10-point category scale with end-word anchors (ranging from dislike—like very much).

Consumer perception of saltiness and purchase intent [43] were also assessed using a category scale with bipolar adjectives on the scale edges, respectively not salty—very salty and highly unlikely—highly likely.

Check-All-That-Apply (CATA)—a multiple-choice consumer-based method using the ‘EsSense25’ lexicon developed by Nestrud et al. [44]—was applied to evaluate product-related emotional associations. Participants were asked to focus on the overall sensory impression of samples and tick all associated emotional expressions from twenty-five that were presented to them in random order. Following Yang et al. [45], the emotions were classified into three categories: positive (active, adventurous, calm, enthusiastic, free, good, good-natured, happy, interested, joyful, loving, nostalgic, pleasant, satisfied, secure, and warm), negative (bored, disgusted, and worried), and unclassified emotional terms (aggressive, guilty, mild, tame, understanding, and wild).

#### 2.5.2. Participants

The study group comprised 105 Irish consumers, the majority of whom were female (67.6%) under the age of 24 (Table 3). The majority of the participants held a third-level degree or higher (49.5%) and were undergraduate students (45.7%).

A substantial percentage of surveyed consumers lived in a big city (64.8%) or an urban town (19.0%). Participants from rural areas (16.2%) were a minority. Participants indicated that their financial position was healthy (59.0%) or okay (36.4%). More than half of the participants (54.3%) ate burgers once or twice a month, and nearly one-fourth (23.8%) ate them three to four times a month. Burgers were most frequently consumed at home, at a friend’s house (50.5%), or at a fast-food establishment (31.4%). Hand-made beef patties (40.9%) and refrigerated patties (44.8%) were the most popular in a study group, with only 14.3% using frozen patties. Close to two-thirds of studied consumers (62.9%) had tasted seaweed before.

The actual Food Neophobia Scores varied from 10 to 57 (max. 70), with higher values corresponding to a higher level of neophobia. The consumers were separated into three groups: the most neophilic (10.0–16.1), the most neutral (16.2–35.0), and the most neophobic (35.1–70.0), with cutoff values established by adding or removing one standard deviation (9.5) from the mean value (25.6).

### 2.6. Statistical Analysis

The IBM SPSS Statistics 27.0.1.0 program (New York, NY, USA) and XLStat 2023 (2023.2.1414, Addinsoft, Paris, France) program were used to investigate the statistical significance of the acquired data. 

To compute the significance of the difference in proximate composition, cooking loss, and WHC, a one-way ANOVA and Tukey’s Honestly Significant Difference (HSD) post hoc test were applied. For the trained panel sensory data, a general linear model (GLM) and Tukey’s HSD were used. Panelists and replicates were treated as random factors, whereas the formulation was specified as a fixed factor. An analysis of covariance was performed using XLSTAT to assess the effect of salt and seaweed content on each dependent variable. Principal component analysis (PCA) was conducted, and a bi-plot was constructed with the first and second PCs projecting formulations and variable scores.

A two-way ANOVA with Fisher Least Significant Differences, wherein formulations are considered fixed effects and consumers are random effects, was used to compare the results of consumer tests. Agglomerative Hierarchical Clustering (AHC) was performed on the collected consumer datasets to structure them into more homogenous subgroups. Student’s *t*-test (numerical data) or the Chi^2^ test (categorical data) was used to evaluate the effect of sociodemographic factors on the liking of seaweed. Ward’s method (Euclidean distance) was used for the agglomeration method in cluster analysis.

Data from CATA (Check-All-That-Apply) were analyzed using the XLSTAT (2023)software tool. Cochran’s Q test (*p* ≤ 0.05) with multiple pairwise comparisons of critical differences (Sheskin) was applied to estimate the significance of differences between study samples. Correspondence analysis (CA) was performed to display the product configuration and determine the relationship between beef patties and emotional expressions. The penalty analysis was performed to relate the degree of liking with the frequency of product-related emotional associations.

## 3. Results

### 3.1. Effect of Sea Spaghetti (Himanthalia elongata) Addition on Proximate Composition of Raw Beef Patties

The proximate analysis of raw burgers showed that salt content ranged between 0.3–1.3% of salt and increased linearly along with increasing seaweed addition (Table 4). The inclusion of 2.5–5.0% of sea spaghetti can successfully replace almost 0.5% of table salt. The addition of 1–2.5% of seaweed to unsalted meat had a lower salt content (0.30–0.33%) than low-salt samples (0.65%).

The cooking loss of the samples decreased with the amount of seaweed added. The samples did not differ significantly in fat, moisture contents, or water-holding capacity (*p* ≥ 0.05).

### 3.2. Effect of Sea Spaghetti (Himanthalia elongata) Incorporation on Sensory Profile in Low-Salt Beef Patties

The addition of seaweed significantly (*p* ≤ 0.05) affected the sensory profile of beef patties (Table 5) for all sensory attributes except tenderness. Sea spaghetti (SW) incorporation significantly decreased the intensity of beef odor and flavor at higher levels of inclusion (1% and 2.5%). Although seaweed odor started to be significantly notable within the level of 2.5% SW, its flavor was recognized when present in a smaller concentration (0.5%). Juiciness was significantly lower in samples with additions of 2.5% seaweed or higher.

The incorporation of 2.5% of seaweed into low-salt burgers (0.5% NaCl) can significantly intensify saltiness (*p* > 0.05) to a similar level as the control sample (1.0% NaCl). On the other hand, the addition of 1–2.5% of SW provided saltiness that was not significantly different from a low-salt sample (0.5% NaCl).

**Figure 2:**
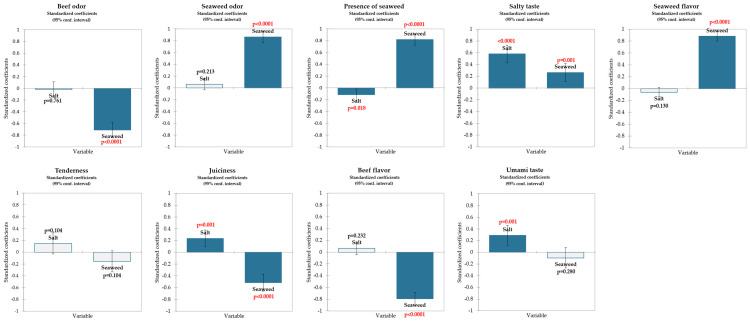
Coefficients from fitted models for the influence of salt content and seaweed content on sensory attributes of beef patties. Dark blue bars indicate a significant effect.

Trained panel sensory data of beef burgers with varying salt (0–1%) and seaweed (0–5%) was subjected to regression analysis. As a result, standardized coefficient charts (Figure 2) that compare the relative magnitude of the effects of different explanatory variables were computed. The beef and seaweed odors and flavors were influenced by the seaweed content. The umami taste was influenced by salt content. Tenderness was not affected by salt or seaweed inclusion. Salt was positively, and seaweed was negatively correlated with juiciness scores.

**Figure 3:**
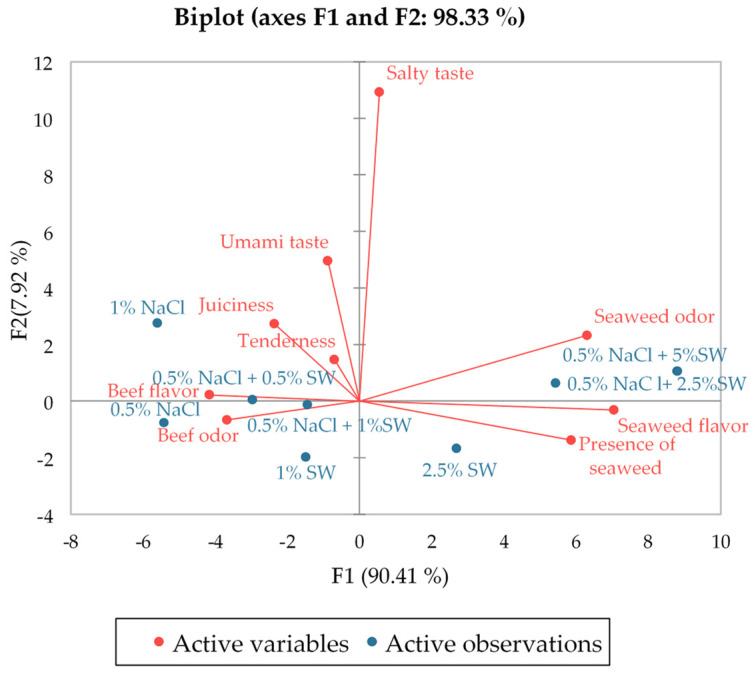
Principal components analysis (PCA) biplot of sensory analysis scores and burger patty formulations with seaweed. NaCl—salt; SW—seaweed.

The first two principal components explained 98.3% of the total variance in sensory scores between samples of burgers with seaweed (Figure 3). The first principal component accounted for 90.4% of the variance, and the biplot demonstrates a close co-location between certain flavor and odor attributes. The intensity of seaweed odor, flavor, and their visual presence in burgers was negatively correlated to the intensity of beef odor and flavor, as well as juiciness, which is demonstrated by the location of those attributes on the opposite side of the F1 origin. The intensity of perception of typical beef characteristics decreases with increasing SW concentration. The second principal component, which explains a smaller proportion of the variance, shows a positive correlation between salty and umami tastes and a smaller influence on juiciness. The angle between the vectors of ‘salty taste’ and ‘presence of seaweed’ approaches a right angle, confirming the independence of these variables.

### 3.3. Effect of Sea Spaghetti (Himanthalia elongata) Incorporation on Consumer Reaction toward Beef Patties

All assessed samples received moderately high hedonic scores (Table 6). Consumer tastings of burgers with seaweed revealed significant differences between the hedonic responses of Irish participants in aroma, appearance, flavor, texture, and overall liking (*p* ≤ 0.05). The aroma-liking scores were similar in the case of all analyzed samples, with the exception of 0.5% NaCl + 2.5% SW, which was less liked. The flavor, texture, and overall liking of samples with 2.5% of seaweed were significantly lower than other samples. The appearance liking was lowered by 2.5% addition of SW, but only in salted samples (S4). The addition of 1% of seaweed did not affect the liking of samples among regular burger consumers.

**Figure 4:**
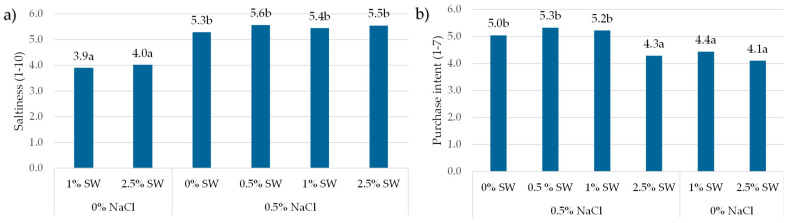
(**a**) Salty taste perception; (**b**) purchase intent of beef burgers with varied seaweed addition in a group of surveyed consumers (*n* = 105) NaCl—salt; SW—seaweed. a, b—mean values marked by different letters above columns, differ significantly at *p* ≤ 0.05. Least Significant Difference, LSD.

Consumer saltiness perception (Figure 4a) of beef burgers without table salt added was significantly lower (3.9–4.0) than in other samples (5.3–5.6). There was no significant difference depending on seaweed level addition. The addition of 2.5% of sea spaghetti causes a statistically significant but relatively minor (20%) reduction in the purchase intent of consumers (Figure 4b).

**Figure 5:**
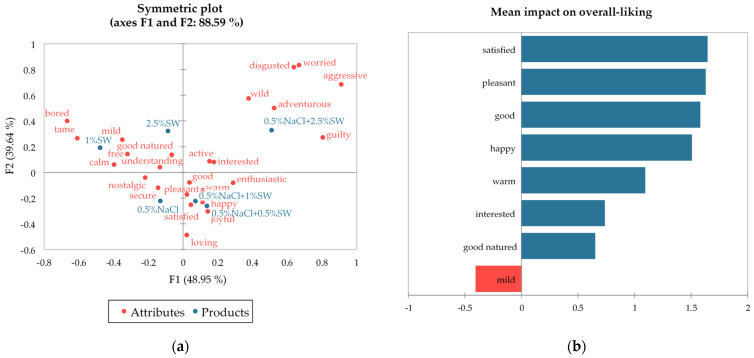
(**a**) Multiple correspondence analysis of CATA responses toward beef patties with seaweed addition (*n* = 105); (**b**) the emotional expression of CATA analysis associated with overall liking. NaCl—salt; SW—seaweed.

In terms of emotional profiling, in general, consumers associated burgers with emotions such as good (29.5–47.6% of total consumers), pleasant (23.8–48.6%), satisfied (21.9–51.4%), and warm (21.9–47.6%), especially in the case of salted samples with up to 1% of seaweed (Table 7, Figure 5a). Interestingly, reduced salty samples with 0.5–1% addition of SW (3.6–3.8 emotions/per consumer) were perceived by consumers as more positive than plain salty samples (3.2), or samples with only seaweed and no salt (2.5–2.6%). Consumption of unsalted burgers aroused more negative emotions (0.3-0.4 emotions/per consumer) than salted ones (0.1–0.2) and was associated with feelings of being bored (17.1–36.2%), mild (31.4–36.2%), and tame (17.1–28.6%). The addition of 2.5% seaweed to salted beef patties was associated with slightly more adventurous (25.7%), but also aggressive (12.4%), and wild (15.2%) emotions.

Figure 5b shows the emotional attributes with a significant mean impact on overall liking. Mean increases are displayed in blue and are identified as “nice to have”. Mean decreases are displayed in red and are identified as must not have. The degree of liking was positively related to the emotions like satisfied, pleasant, good, and happy and negatively related to mild.

To investigate the factors affecting the lower overall liking of samples with higher seaweed addition (0.5%NaCl + 2.5 % SW and 2.5% SW) and divide consumers into more homogeneous subgroups, the agglomeration method of cluster analysis was applied. The participants belonged to two clusters:

**Cluster 1** (*Seaweed enthusiasts, n = 75*, *71.4% of participants*)—consumers who are more eager to eat meat products with seaweed additions. The consumer group consisted of a majority of women, with a higher number of men and people between the ages of 25 and 44, more people with a third-level degree or higher, and people from bigger cities. A higher share of consumers declared previous experience with seaweed. An average representative of this group has a good impression of edible seaweed and is optimistic about food with SW.

**Figure 6:**
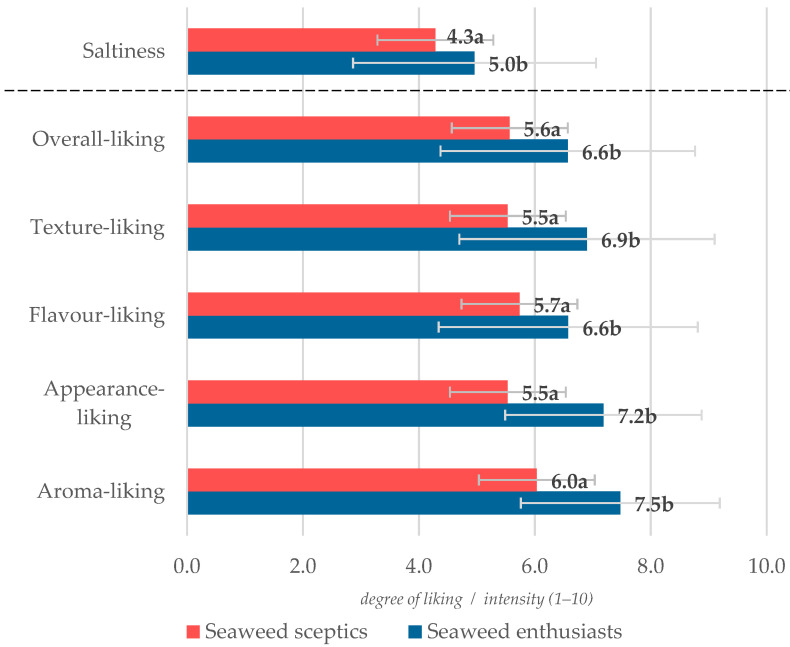
Degree of liking and saltiness perception samples with 2.5% seaweed addition in a group of seaweed enthusiasts and seaweed skeptics. a, b—mean values marked by different letters next to columns, differ significantly at *p* ≤ 0.05.

**Cluster 2** (*Seaweed skeptics*, *n = 30, 28.6% of participants*)—consumers that are mainly young women, declaring a healthy financial situation, some of them living in rural areas. This group of consumers was represented by the most neophobic subjects (mean of FNS = 37.8) who tried to avoid foods with additives. This group of consumers assessed the liking of burgers as significantly lower, as well as the perceived saltiness of beef patties (Figure 6). Seaweed skeptics were also less inclined to express their emotions, especially those positive ones, in the CATA assessment. These participants less frequently perceived them as free, good, happy, pleasant, and warm. On the other hand, they have seen them as more guilty.

## 4. Discussion

The addition of seaweed to foods provides nutritional, technological, and sensory functionality [46]. However, at high inclusion levels, sensory quality may be a limitation of their application. Generally, various seaweeds have intense marine, sea, wild mushroom, or crustacean flavors. In the case of sea spaghetti, it evokes a soft, land vegetable flavor [47]. For this reason, the study aimed to explore the possibility of sea spaghetti application in the production of low-salt beef patties and its effect on sensory profile and hedonic emotional reaction.

This study revealed that the intensity of seaweed odor, flavor, and visual presence in burgers are negatively related to the intensity of beef odor, flavor, and juiciness, with typical beef characteristics decreasing with increasing SW concentration. Our findings are similar to the results of other authors [15,16,48,49] in terms of texture and flavor deterioration, who found that the addition of macroalgae in sausages causes harder texture and higher off-flavor intensity. López-López et al. [48] found that the addition of 5% of sea spaghetti significantly increased the off-flavor intensity. They suggest that panelists were unaccustomed to the distinctive flavor of seaweed, which can be limited by selecting less strongly flavored seaweed or reformulating the seasoning. A recent study [22] revealed that the sensory profile of reformulated frankfurters containing seaweed was greatly influenced by the type of added seaweed, and *Himanthalia elongata* was the most accepted. Vilar et al. [22] reported that the addition of 1% of sea spaghetti to frankfurters significantly decreased meat flavor, increased seaweed flavor, and off-flavor intensities, similar to our study. Meat flavor intensity may drop independently as a result of salt reduction, as proved by the study of Carvalho et al. [50]. Seaweed also affects texture attributes, as Kim et al. [49] found that the addition of dried sea tangle significantly reduces juiciness and lowers the tenderness of breakfast sausages at a level of 4%. Edible seaweed types also have varied effects on juiciness and hardness. *Himanthalia elongata* did not deteriorate texture (juiciness, tenderness) at a level of 1%. In this study, even high SW inclusion levels did not affect tenderness, while juiciness significantly decreased at 2.5%. This is probably related to the absorption of moisture from comminuted meat matrices by seaweed.

The addition of seaweed reduced cooking loss compared to a control sample. With increasing inclusion levels of seaweed, the cooking loss decreased; however, a significant reduction was noticed at the level of 2.5% of SW. The results are consistent with previous results by López-López et al. [26,27], who found that the addition of seaweed to beef patties can significantly limit the cooking loss during the preparation day or over the storage time. Similar results were reported on pork meat patties [51] and sausages [14,48,52]. Cox & Abu-Ghannam [28] found that the addition of blanched sea spaghetti to the patties reduced the cooking losses. Sea spaghetti added to comminuted pork products at a level of 2.5–5.0% [13,14] and to low-fat frankfurters at a level of 5.6% [53] were effective structuring agents that led to the formation of harder and chewier, more heterogeneous structures with better water and fat binding properties [12]. This phenomenon may result from the binding functionality of main seaweed components, like hydrocolloids, dietary fiber, and minerals. Gómez-Ordóñez et al. [54] reported that the swelling and water retention capacity of edible seaweed were high, while fat retention was low due to the hydrophilic nature of fiber polysaccharides.

In our study, seaweed addition to raw beef patties did not affect moisture, fat, or protein content. In the case of beef products, seaweeds have been added in various formats, such as dried powder, fresh, and oil. The results of [25] revealed that Chlorella and Spirulina proteins could be useful in new meat products. The 1% inclusion of microalgae increases the concentrations of all amino acids in beef patties. In a study by López-López [48], the addition of 5% of sea spaghetti did not change the protein content but lowered moisture and increased ash content in reformulated frankfurters.

Seaweed, rich in potassium and sodium, has a naturally salty taste and can be used as a healthy sodium substitute in food [55]. Our findings showed that the addition of 2.5% seaweed to low-salt burgers can increase the perception of saltiness to a similar level to the control (1% NaCl) sample. The use of 1–2.5% SW instead of table salt can leverage a saltiness that is not significantly different from a low-salt sample, as according to analytical measurement, the addition of 2.5–5.0% sea spaghetti can effectively substitute about 0.5% of the table salt. Seaweed bread with 10% and 20% salt reduction was acceptable to consumers, but increased salt reduction led to negative flavor and textural attributes [56].

In this study, the saltiness derived from seaweed was perceived by trained panelists but not by consumers, who indicated that the perceived saltiness of unsalted samples with seaweed addition was significantly lower than that of the low-salt sample.

A study by Notowidjojo et al. [57] showed that a 1:1 ratio between red macroalgae *Euchema cottoni* and table salt is the most acceptable when blended with seaweed salt. The seaweed salt product exhibited significantly higher potassium and magnesium content than ordinary salt, as well as lower sodium and iodine content. The sea spaghetti used in this study had 12.7% salt [19], hence salt sourced from various levels of seaweed addition (0.5–5%) ranged from 0.06–0.64 g. Nevertheless, seaweed-derived saltiness should not be interpreted one-to-one with the salt content. Salt could be trapped in the algae structure and have different release dynamics. Saltiness perception may not only arise mainly from sodium and potassium but also from odor-induced saltiness enhancement or interaction between salty and sour tastes. The saltiness of the samples could also be attributed to the high content of glutamate and 5’ ribonucleotides in seaweed, which enhances the umami flavor in Japanese dishes. It also contains halogenated compounds contributing to marine and shellfish aromas [47,58]. In our study, seaweed content was not related to umami taste intensity.

Our findings revealed that the addition of higher levels of seaweed decreased the liking of low-salt (0.5% NaCl) patties (at a seaweed inclusion level of 2.5%), while samples with 0.5 and 1.0% of SW did not differ in consumer liking from the low-salt formulations. Incorporating 1% of seaweed into unsalted beef patties did not negatively affect the liking of samples among regular burger consumers. Interestingly, a low addition of seaweed (<1%) does not significantly lower the purchase intent and thus might be perceived by some consumers as a desired ingredient. However, purchase intention in samples only with seaweed and at the highest inclusion level has dropped by around 20%.

In general, the addition of seaweed to sausages lowers overall acceptability [15,16,48,49]. A study by Vilar et al. [22] revealed that the overall acceptability of frankfurters was lower when 1% of *Undaria pinnatifolia*, *Porphyra umbilicalis*, and *Palmaria *palmata** were added, whereas in the case of *Himanthalia elongata,* it did not differ significantly from the control sample. According to Mahammed et al. [16], the overall sensory acceptability of cooked sausage did not decrease (*p* < 0.05) at 2.5% of the sea spaghetti addition, and a significant difference appeared at the level of inclusion of 5%. This supports the early results of the López-López et al. [48] study, which showed an acceptability decrease in frankfurters with 5% of *Himanthalia elongata* inclusion.

In our study, a lower level of processing meat compared with typical sausages was used, while the type of seaweed (fresh, blanched, and dried) and its comminution (shredded, powdered) level may have had an effect. Some studies conducted with small groups of consumers suggest a higher acceptability of seaweed burgers than the control sample. A study by Cox and Abu-Ghannam [28] showed that the addition of blanched seaweed to beef patties outperformed the control sample in flavor and overall acceptability. The consumer acceptability of seaweed samples could be higher than the control sample, probably due to the removal of the seaweed odor during the blanching process. Similarly, in the study of Pindi et al. [59], the chicken patty with 1.5% salt and 4% seaweed had the highest overall acceptability, while the patty with 1% salt and 4% seaweed had significantly higher acceptability than the control sample.

Acceptance of products with seaweed depends on cultural circumstances and culinary heritage but also arises from the congruity of food matrices added to them. Seaweed has been eaten in the diets of coastal cultures for centuries. The results of Wendin and Undeland [60] showed that Swedish consumers would like to have seaweed added to snacks, spices, dishes, and bread. Norwegian consumers’ attitudes towards seaweed food products are influenced by biospheric values and beliefs about their health and naturalness, with perceived uniqueness playing a significant role [61]. Lamont and McSweeney [62] demonstrated the acceptability of seaweed in a Western population, which may encourage and promote their consumption by adding seaweed to bread in small amounts. Canadian consumers were more likely to purchase dried seaweed and seaweed-added bread than other food items (*p* < 0.05). Participants also indicated a preference for seaweed in fish filets, cheese, and beef burgers over yogurt and sausages [63].

The acceptance of seaweed burgers may arise from the individual characteristics of the subjects. Our study revealed that the most reluctant consumers (a cluster of seaweed skeptics), who liked samples with a 2.5% addition of sea spaghetti significantly less, were the most neophobic subjects and avoided a range of additives in food. They expressed fewer emotions in the CATA assessment and were viewed less positively and more guilty, with less perceived freedom, happiness, and warmth. Our findings are supported by the research of Losada-Lopez et al. [24], who found that neophobia affects consumer attitudes towards seaweed and reduces willingness to consume it. Seaweed is not easily accepted if it is not part of the culinary heritage. The Italian study of Palmieri and Forleo [64] found that 76% of the surveyed group were willing to eat seaweed, possibly due to familiarity with traditional Italian or Asian dishes. However, only 57% had eaten seaweed before. In our study, slightly more (62.9%) participants declared previous seaweed tastings. Seaweed consumption in Ireland has re-emerged, gaining popularity as a nutritious and versatile food and adding flavor to innovative dishes [65,66]. On the other hand, food neophobia did not affect the purchase intent or emotional responses of Canadian consumers assessing their attitudes toward food containing seaweed [63]. The study of Palmieri et al. [67] found a positive correlation between perceived environmental impact and previous tasting experiences on seaweed perception, which was also exhibited in our study by extracting from a group of consumer clusters of seaweed enthusiasts who significantly preferred the burgers with 2.5% SW addition. Understanding consumer behavior and facilitating information-based decisions can reduce skepticism and promote a sustainable seaweed food industry [68].

Consumers generally rated tested burgers positively (i.e., good, pleasant, satisfied, and warm), particularly salted samples with up to 1% seaweed. The 2.5% addition of seaweed to salted beef patties was slightly more adventurous, aggressive, and wild. Unsalted burgers with seaweed (1–2.5%) evoked more negative emotions and were associated with being bored, mild, and tame. Similarly, in the study of Moss and McSweeney [63], beef burgers containing seaweed were found to be linked to negative emotions such as boredom, disgust, worry, aggression, and guilt. Although the beef burger was associated with negative emotions, the participants rated their purchase intent for beef burgers moderately high.

## 5. Conclusions

Seaweed (SW) significantly impacted the sensory profile of beef patties, decreasing beef odor and flavor intensity. Seaweed odor and flavor were negatively related to beef odor and flavor intensity. Incorporating 2.5–5% seaweed intensified saltiness in low-salt burgers, while 0.5–1% SW provided similar saltiness. One percent sea spaghetti seaweed can be successfully applied to low-salt beef patties, bringing hedonic and emotional benefits without a significant salt content increase.

Irish participants’ liking for seaweed in burgers varied among samples, with an inclusion level of 2.5% negatively affecting the flavor, texture, and overall liking. Consumers noticed a lack of saltiness in unsalted samples of sea spaghetti. The inclusion of 0.5–1% of SW in low-salt samples may be perceived by consumers as desired in terms of hedonic and emotional reactions, as it evokes more positive emotions compared to samples with only added salt or samples with only added seaweed. The liking was positively related to emotions like satisfaction, pleasure, good, and happiness and negatively related to mild. The consumption of unsalted burgers with seaweed (1–2.5%) elicited more negative emotions and was linked to feelings of being bored, mild, and tame. The cluster analysis extracted two groups of consumers, i.e., seaweed enthusiasts and seaweed skeptics with high food neophobia and avoidance of foods with additives, who less liked burgers with 2.5% seaweed addition, perceived them as less salty, and were less inclined to express their emotions, especially these positive ones.

## Figures and Tables

**Table 1:** Formulations of burgers with seaweed.

ID	Formulation	Meat	Salt	Sea Spaghetti
**CS**	1% NaCl	99.0	1.0	0.0
**S1**	0.5% NaCl	99.5	0.5	0.0
**S2**	0.5% NaCl + 0.5% SW	99.0	0.5	0.5
**S3**	0.5% NaCl + 1% SW	98.5	0.5	1.0
**S4**	0.5% NaCl + 2.5% SW	97.0	0.5	2.5
**S5**	0.5% NaCl + 5% SW	94.5	0.5	5.0
**S6**	1% SW	99.0	0.0	1.0
**S7**	2.5% SW	97.5	0.0	2.5

CS—control sample; S—sample; NaCl—salt; SW—seaweed.

**Table 2:** List of sensory attributes, lexicon, and reference materials used in sensory profiling.

Sensory Attribute	Definition	Scale	Reference Material
**Odor**			
Beef odor	The amount of beef odor in the sample	none—very strong	**Weak:** beef patty boiled in plenty of water to reach 75 °C in the core; **Medium:** beef patty cooked in a microwave oven to reach 75 °C; **Strong:** beef patty grilled to reach 75 °C in the core
Seaweed odor	Characteristic smell of seaweed, wet, earthy, green, marine	none—very strong	**Very weak:** no seaweed burger; **Weak:** beef burger with the addition of 1% of seaweed **Medium:** beef burger with the addition of 5% of seaweed; **High:** beef burger with the addition of 10% of seaweed **Very high:** samples of fresh seaweed
**Appearance**			
Presence of seaweed	the visible presence of seaweed in the sample	none—very strong	**Low:** no seaweed burger; **Medium:** beef burger with the addition of 5% of seaweed; **High:** beef burger with the addition of 10% of seaweed
**Texture**			
Tenderness	force required to bite the sample with molar teeth during the first bite	not tender—very tender	**Low:** beef patty grilled to reach 95 °C in the core **Medium:** beef patty grilled to reach 75 °C in the core **High:** beef patty grilled to reach 71 °C in the core
Juiciness	amount of liquid released from sample during chewing	not juicy—very juicy	
**Flavor/taste**			
Beef flavor	Intensity of beef flavor in the sample	none—very strong	**Weak:** beef patty boiled in plenty of water to reach 75 °C in the core; **Medium:** beef patty cooked in a microwave oven to reach 75 °C; **Strong:** beef patty grilled to reach 75 °C in the core
Salty taste	The level of saltiness in the sample	none—very strong	**Very weak:** no salt burger patties **Weak:** burger patty with 0.5% NaCl; **Medium:** burger patty with 0.8% NaCl; **Strong:** burger patty with 1.6% NaCl
Umami taste	The level of umami taste in the sample	none—very strong	**Very weak:** 0.056% MSG aqueous solution; **Weak:** no MSG burger; **Medium:** beef burger with the addition of 0.5% of MSG; **High:** beef burger with the addition of 1.0% of MSG
Seaweed flavor	Characteristic flavor of seaweed	none—very strong	**Weak:** no seaweed burger; **Medium:** beef burger with the addition of 5% of seaweed; **High:** beef burger with the addition of 10% of seaweed

**Table 3:** Characteristics of the study group (*n* = 105).

Feature *	Group	Participants	
		Number (*n*)	Percentage (%)
Total		105	100.0
Gender	women	71	67.6
	men	34	32.4
Age	19–24 years	78	74.3
	25–44 years	24	22.9
	45–65 years	2	1.9
	65 years and over	1	1.0
Education	upper secondary	5	4.8
	third level non-degree	48	45.7
	third-level degree and higher	52	49.5
Residence place	city	68	64.8
	urban town	20	19.0
	rural area	17	16.2
Financial situation, in own opinion	healthy	62	59.0
	OK	38	36.2
	tight	5	4.8
Burger consumption’ frequency	less than once per month	4	3.8
	once or twice a month	57	54.3
	three-four times a month	25	23.8
	once a week	15	14.3
	two or three times a week	4	3.8
Burger consumption venue	At my home or a friend’s house	53	50.5
	fast-food outlet	33	31.4
	gourmet burger outlet	14	13.4
	street-food vendors	6	3.8
	other food service venue or place	1	1.0
Types of used burgers	hand-made (prepared from scratch)	45	42.9
	refrigerated beef patties	46	43.8
	frozen beef patties	14	13.3
Food Neophobia Level	the most neophiliac (10.0–16.1)	70	66.7
	the most neutral (16.2–35.0)	16	15.2
	the most neophobic (35.1–70.0)	19	18.1
Level of habitual dietary salt intake	average salt intake	55	52.4
	higher salt intake	50	47.6
Previous tasting of seaweed	Yes	66	62.9
	No	39	37.1

* Stratification of socio-demographic features by the Central Statistics Office in Ireland.

**Table 4:** Technological properties of raw beef patties with seaweed.

ID		Formulation	Cook Loss	WHC	Moisture	Protein Content	Fat Content	Salt Content
	NaCl	Seaweed	$\mathrm{percentage}\;(\%)\;\bar{x}$ ± SD					
**CS**	1%	-	23.70 ^a^ ± 2.08	80.48 ^a^ ± 6.46	66.70 ^a^ ± 1.03	33.07 ^a^ ± 0.66	13.48 ^a^ ± 0.34	1.04 ^d^ ± 0.03
**S1**	0.5%	-	24.16 ^a^ ± 0.26	79.49 ^a^ ± 3.35	66.19 ^a^ ± 0.68	35.13 ^a,b^ ± 0.85	13.04 ^a^ ± 1.83	0.56 ^b,c^ ± 0.08
**S2**	0.5%	0.5%	21.02 ^a,b^ ± 1.44	87.25 ^a^ ± 6.25	66.57 ^a^ ± 1.36	33.50 ^a^ ± 0.88	12.38 ^a^ ± 0.84	0.61 ^b^ ± 0.09
**S3**	0.5%	1%	19.78 ^a,b,c^ ± 0.69	89.01 ^a^ ± 0.57	65.44 ^a^ ± 2.30	35.99 ^b^ ± 0.27	14.80 ^a^ ± 0.88	0.64 ^b,c^ ± 0.05
**S4**	0.5%	2.5%	14.56 ^c,d^ ± 0.44	91.60 ^a^ ± 0.99	62.75 ^a^ ± 4.07	35.38 ^a,b^ ± 2.10	14.35 ^a^ ± 2.26	0.88 ^c^ ± 0.12
**S5**	0.5%	5%	8.00 ^b,c^ ± 1.49	87.57 ^a^ ± 1.00	65.81 ^a^ ± 1.21	34.13 ^a,b^ ± 1.32	13.04 ^a^ ± 0.48	1.26 ^d^ ± 0.09
**S6**	-	1%	19.60 ^a,b,c^ ± 2.00	83.83 ^a^ ± 7.74	65.46 ^a^ ± 0.94	34.64 ^a,b^ ± 0.77	14.34 ^a^ ± 0.65	0.30 ^a^ ± 0.11
**S7**	-	2.5%	17.00 ^b,c^ ± 3.20	85.61 ^a^ ± 5.29	63.77 ^a^ ± 1.46	36.10 ^b^ ± 0.52	14.13 ^a^ ± 1.09	0.33 ^a^ ± 0.06

^a,b,c,d^—least squares mean values in columns that do not share a common superscript differ (*p* ≤ 0.05). NaCl—salt; SW—seaweed.

**Table 5:** Sensory profile of beef patties with varied concentrations of seaweed.

Sample		Beef Odor	Seaweed Odor	Presence of Seaweed	Tenderness	Juiciness	Beef Flavor	Salty Taste	Umami Taste	Seaweed Flavor
		$\bar{x}$ **Intensity (0–10) ± SE**								
**CS**	1% NaCl	6.3 ^a^ ± 0.3	0.3 ^d^ ± 0.1	0.1 ^e^ ± 0.1	6.7 ^a^ ± 0.3	6.9 ^a^ ± 0.1	6.7 ^a^ ± 0.2	5.9 ^a^ ± 0.4	5.6 ^a^ ± 0.4	0.1 ^e^ ± 0.0
**S1**	0.5% NaCl	5.9 ^a^ ± 0.4	0.2 ^d^ ± 0.1	0.2 ^e^ ± 0.1	6.5 ^a^ ± 0.4	6.0 ^a^ ± 0.3	6.9 ^a^ ± 0.2	2.9 ^c,d^ ± 0.5	3.7 ^a,b^ ± 0.4	0.1 ^e^ ± 0.0
**S2**	0.5% NaCl + 0.5% SW	5.7 ^a,b^ ± 0.3	1.2 ^d^ ± 0.2	1.9 ^d^ ± 0.3	6.3 ^a^ ± 0.4	5.9 ^a,b^ ± 0.2	6.2 ^a,b^ ± 0.2	3.6 ^b,c,d^ ± 0.5	4.5 ^a,b^ ± 0.4	1.5 ^d^ ± 0.3
**S3**	0.5% NaCl + 1% SW	5.3 ^a,b^ ± 0.3	1.7 ^d^ ± 0.3	2.6 ^d^ ± 0.3	6.3 ^a^ ± 0.3	5.6 ^a,b,c^ ± 0.3	5.4 ^b^ ± 0.3	3.5 ^b,c,d^ ± 0.5	4.4 ^a,b^ ± 0.4	2.6 ^d^ ± 0.3
**S4**	0.5% NaCl+ 2.5% SW	3.3 ^c,d^ ± 0.4	5.3 ^b^ ± 0.6	5.5 ^b^ ± 0.04	6.0 ^a^ ± 0.2	4.4 ^c,d^ ± 0.3	3.2 ^c,d^ ± 0.3	4.6 ^a,b,c^ ± 0.5	4.0 ^a,b^ ± 0.6	6.4 ^b^ ± 0.3
**S5**	0.5% NaCl + 5% SW	1.8 ^d^ ± 0.3	7.5 ^a^ ± 0.4	7.0 ^a^ ± 0.3	5.7 ^a^ ± 0.4	3.7 ^d^ ± 0.3	2.3 ^d^ ± 0.3	5.0 ^a,b^ ± 0.5	3.9 ^a,b^ ± 0.4	7.6 ^a^ ± 0.2
**S6**	1% SW	5.2 ^a,b^ ± 0.3	1.5 ^d^ ± 0.01	3.1 ^c,d^ ± 0.1	5.6 ^a^ ± 0.3	4.7 ^b,c,d^ ± 0.1	5.5 ^b^ ± 0.2	2.0 ^d^ ± 0.4	3.8 ^a,b^ ± 0.4	1.9 ^d^ ± 0.0
**S7**	2.5% SW	4.2 ^b,c^ ± 0.3	3.6 ^c^ ± 0.5	4.0 ^c^ ± 0.4	6.1 ^a^ ± 0.3	4.7 ^b,c,d^ ± 0.4	3.8 ^c^ ± 0.3	2.4 ^d^ ± 0.2	3.1 ^b^ ± 0.4	5.2 ^c^ ± 0.4

^a,b,c,d,e^—mean values marked by different letters in columns differ significantly at *p* ≤ 0.05. NaCl—salt; SW—seaweed.

**Table 6:** Consumer (*n* = 105) hedonic scores for beef patty formulations with seaweed and salt.

Sample		Aroma	Appearance	Flavor	Texture	Overall Liking
		Mean Value of Hedonic Scores (1–10) ± SE				
S1	0.5% NaCl	7.4 ^b^ ± 0.2	7.3 ^b^ ± 0.2	7.8 ^b^ ± 0.2	7.2 ^b^ ± 0.2	7.5 ^b^ ± 0.2
S2	0.5% NaCl + 0.5% SW	7.4 ^b^ ± 0.2	7.3 ^b^ ± 0.2	7.8 ^b^ ± 0.2	7.5 ^b^ ± 0.2	7.7 ^b^ ± 0.2
S3	0.5% NaCl + 1% SW	7.5 ^b^ ± 0.2	7.3 ^b^ ± 0.2	7.7 ^b^ ± 0.2	7.3 ^b^ ± 0.2	7.5 ^b^ ± 0.2
S4	0.5% NaCl + 2.5% SW	6.8 ^a^ ± 0.2	6.5 ^a^ ± 0.2	6.6 ^a^ ± 0.2	6.6 ^a^ ± 0.2	6.4 ^a^ ± 0.2
S6	1% SW	7.4 ^b^ ± 0.2	7.1 ^b^ ± 0.2	6.7 ^a^ ± 0.2	6.9 ^a,b^ ± 0.2	6.7 ^a,b^ ± 0.2
S7	2.5% SW	7.3 ^b^ ± 0.2	6.9 ^a,b^ ± 0.2	6.1 ^a^ ± 0.2	6.4 ^a^± 0.2	6.1 ^a^ ± 0.2

^a,b^—mean values marked by different letters in columns differ significantly at *p* ≤ 0.05 Least Significant Difference, LSD. NaCl—salt; SW—seaweed.

**Table 7:** Emotional associations of beef burgers with varied seaweed additions (*n* = 105).

EsSense25 Product-Related Associations	Beef Burgers with Varied Seaweed Addition						
	0.5% NaCl				0% NaCl		*p*-Values
	0% SW	0.5% SW	1% SW	2.5% SW	1% SW	2.5% SW	
	Percent of Indications in the Group of Surveyed Consumers (%)						
active	1.9 ^a^	10.5 ^a^	5.7 ^a^	5.7 ^a^	4.8 ^a^	7.6 ^a^	NS
adventurous	3.8 ^a^	8.6 ^a^	10.5 ^a,b^	25.7 ^b^	5.7 ^a^	15.2 ^a b^	<0.0001
aggressive	1.0 ^a^	3.8 ^a,b^	1.0 ^a^	12.4 ^b^	1.0 ^a^	3.8 ^a b^	<0.0001
bored	7.6 ^a^	5.7 ^a^	10.5 ^a^	4.8 ^a^	36.2 ^b^	17.1 ^a^	<0.0001
calm	22.9 ^b^	14.3 ^a,b^	12.4 ^a b^	5.7 ^a^	26.7 ^b^	19.0 ^a b^	0.000
disgusted	0.0 ^a^	1.0 ^a,b^	1.9 ^a,b^	6.7 ^b^	1.0 ^a,b^	4.8 ^a,b^	0.010
enthusiastic	8.6 ^a,b^	15.2 ^b^	13.3 ^a,b^	11.4 ^a,b^	3.8 ^a^	9.5 ^a,b^	0.056 *
free	5.7 ^a^	2.9 ^a^	6.7 ^a^	2.9 ^a^	8.6 ^a^	6.7 ^a^	NS
good	37.1 ^a,b^	47.6 ^b^	42.9 ^a,b^	30.5 ^a,b^	32.4 ^a,b^	29.5 ^a^	0.010
good-natured	15.2 ^a^	21.0 ^a^	20.0 ^a^	17.1 ^a^	23.8 ^a^	22.9 ^a^	NS
guilty	1.9 ^a^	1.9 ^a^	1.0 ^a^	4.8 ^a^	0.0 ^a^	1.0 ^a^	NS
happy	26.7 ^a,b,c^	38.1 ^b,c^	41.0 ^c^	21.9 ^a,b^	20.0 ^a^	13.3 ^a^	<0.0001
interested	16.2 ^a^	29.5 ^a^	30.5 ^a^	29.5 ^a^	20.0 ^a^	23.8 ^a^	0.041
joyful	14.3 ^a,b^	21.0 ^b^	21.0 ^b^	10.5 ^a,b^	8.6 ^a,b^	5.7 ^a^	0.001
loving	10.5 ^a,b^	17.1 ^b^	11.4 ^a,b^	2.9 ^a^	5.7 ^a,b^	2.9 ^a^	0.000
mild	21.9 ^a,b,c^	12.4 ^a^	19.0 ^a,b^	12.4 ^a^	36.2 ^c^	31.4 ^b,c^	<0.0001
nostalgic	11.4 ^a^	8.6 ^a^	2.9 ^a^	2.9 ^a^	6.7 ^a^	8.6 ^a^	0.052 *
pleasant	47.6 ^b^	48.6 ^b^	47.6 ^b^	29.5 ^a^	32.4 ^a,b^	23.8 ^a^	<0.0001
satisfied	40.0 ^b,c^	45.7 ^c^	51.4 ^c^	21.9 ^a^	23.8 ^a,b^	21.9 ^a^	<0.0001
secure	23.8 ^b^	16.2 ^a,b^	15.2 ^a,b^	8.6 ^a^	15.2 ^a,b^	13.3 ^a,b^	0.037
tame	16.2 ^a,b,c^	6.7 ^a,b^	6.7 ^a,b^	3.8 ^a^	28.6 ^c^	17.1 ^b,c^	<0.0001
understanding	4.8 ^a^	6.7 ^a^	4.8 ^a^	2.9 ^a^	5.7 ^a^	6.7 ^a^	NS
warm	43.8 ^b^	47.6 ^b^	38.1 ^a,b^	31.4 ^a,b^	21.9 ^a^	26.7 ^a^	<0.0001
wild	2.9 ^a^	4.8 ^a^	4.8 ^a^	15.2 ^b^	5.7 ^a,b^	10.5 ^a,b^	0.002
worried	1.0 ^a^	1.9 ^a^	1.0 ^a^	9.5 ^b^	1.0 ^a^	6.7 ^a,b^	0.001
Average number of emotions per consumer							
**positive**	3.2	3.8	3.6	2.6	2.5	2.5	
**negative**	0.1	0.1	0.1	0.2	0.4	0.3	
**unclassified**	0.5	0.4	0.4	0.5	0.8	0.7	
**Total emotions**	3.9	4.4	4.2	3.3	3.8	3.5	

Citation frequencies marked by different letters in rows differ significantly. Multiple pairwise comparisons using the critical difference (Sheskin) procedure were applied. NS—Not significant. * statistical trend toward significance. NaCl—salt; SW—seaweed.

## Data Availability

The original contributions presented in the study are included in the article/Supplementary Material, further inquiries can be directed to the corresponding authors.

## Supplementary Materials

The following supporting information can be downloaded at: https://www.mdpi.com/article/10.3390/foods13081197/s1, Figure S1: Photograph of raw burgers used in the study; File S1: Questionnaire used in consumer tests.
