# Supplementary material for: Incorporation of Sea Spaghetti (Himanthalia elongata) in Low-Salt Beef Patties: Effect on Sensory Profile and Consumer Hedonic and Emotional Response
Source: Foods. 2024 Apr 15;13(8):1197. doi: 10.3390/foods13081197 (PMC11049442; doi:10.3390/foods13081197)
Supplement: Supplementary file 1 [file foods-13-01197-s001.zip › File S1 Questionnaire used in consumer tests.pdf]

First session
Participant no.
____

Thank you for accepting invitation for the burger testing
We really appreciate your opinion

You are taking part in a consumer tasting of beef burgers. Please begin the testing by writing your participant number, completing a first part of the survey, and then proceed to the next section. Please use a blue pen to fill in the form and write crosses in boxes and on appropriate place on scale e.g.

Question:
☒ answer a
☐ answer b

very weak ○ ○ ○ ○ ⊗ ○ ○ ○ ○ ○ very strong

[X] answer a [ ] answer b [X] answer c

What is your gender?
[ ] female
[ ] male
[ ] non-binary
[ ] prefer not to disclose

What is your age?
[ ] 19 – 24 years
[ ] 25 – 44 years
[ ] 45 – 65 years
[ ] 65 years and over

Please specify your age: ______

What is your education level?
[ ] no formal/primary/lower secondary
[ ] upper secondary
[ ] third level non degree
[ ] third level degree and higher

What is your place of residence?
[ ] city (population greater than 50,000)
[ ] urban town (population between 1,500 and 49,999)
[ ] rural area (population lower then 1,500)

Financial situation:
[ ] healthy (I have money left at the end of the month for a few luxuries or to add to my savings)
[ ] OK (I get by, but there's not a lot left by the time the basics are taken care of)
[ ] tight – I'm making ends meet, but only just
[ ] struggling – I'm in danger of falling behind with bills or loan repayments
[ ] in trouble

How often do you eat the beef burgers?
[ ] never
[ ] once or two a month
[ ] three-four times a month
[ ] once a week
[ ] two or three times a week
[ ] every day

Where do you usually eat beef burgers?
[ ] at my home or at friends' house
[ ] fast-food outlet
[ ] gourmet burger outlet
[ ] street-food vendors
[ ] other foodservice venue or place (please specify ………..)

When cooking burgers, what type of burgers do you usually use?
[ ] hand-made (prepared from scratch),
[ ] refrigerated beef patties
[ ] frozen beef patties

Have you ever tried seaweed before?
[ ] no, I have not
[ ] yes, I have

How often do you use salt at the table?
[ ] never or rarely
[ ] quite often
[ ] always or very often

How much bread do you eat in one day?
[ ] always unsalted bread or bread
[ ] with very little salt
[ ] 3 or less than 3 slices of bread a day
[ ] ≥ 4-5 slices of bread for day

How many times a week do you eat cheese and/or cold cuts?
[ ] 0-2 times a week
[ ] 3-4 times a week
[ ] 5 or more times a week

Do you ever get thirsty, especially after a meal?
[ ] never or rarely
[ ] quite often
[ ] always or very often

When you eat out, food seems usually?
[ ] salty
[ ] normal
[ ] insipid

How strong is your desire to eat right now?

very weak ○ ○ ○ ○ ○ ○ ○ ○ ○ ○ very strong

To what extent do you agree with the following statements? *please mark X in the appropriate box*

Statements	strongly disagree	disagree	more or less disagree	undecided	more or less agree	agree	strongly agree
	1	2	3	4	5	6	7
1. I am constantly sampling new and different cuisines.	◯	◯	◯	◯	◯	◯	◯
2. I do not trust new foods.	◯	◯	◯	◯	◯	◯	◯
3. If I do not know what is in a food, I will not try it.	◯	◯	◯	◯	◯	◯	◯
4. I like foods from different countries.	◯	◯	◯	◯	◯	◯	◯
5. Food that I have not eaten before, look too weird to eat.	◯	◯	◯	◯	◯	◯	◯
6. At dinner parties I will try a new food.	◯	◯	◯	◯	◯	◯	◯
7. I am afraid to eat things I have never had before.	◯	◯	◯	◯	◯	◯	◯
8. I am very particular about the foods I will eat.	◯	◯	◯	◯	◯	◯	◯
9. I will eat almost anything.	◯	◯	◯	◯	◯	◯	◯
10. I like to try restaurants with new cuisines.	◯	◯	◯	◯	◯	◯	◯
11. My overall impression of edible seaweeds is good.	◯	◯	◯	◯	◯	◯	◯
12. I am certain about the quality and safety of edible seaweed for consumption.	◯	◯	◯	◯	◯	◯	◯
13. I am optimistic about the overall quality of food containing seaweeds and I will buy in the future.	◯	◯	◯	◯	◯	◯	◯
14. I try to avoid food products with additives.	◯	◯	◯	◯	◯	◯	◯
15. To me the naturalness of the food that I buy is an important quality.	◯	◯	◯	◯	◯	◯	◯
16. When I eat out my menu choices are based on healthiness of the meals	◯	◯	◯	◯	◯	◯	◯
17. I would rather eat a smaller portion of my favourite meal in a fast food restaurant than a reduced calorie version.	◯	◯	◯	◯	◯	◯	◯

During this session, you will receive **6 samples**, each of which is a quarter of a burger.

Testing instruction

1. You will be receiving samples of burgers one by one. Take a sip of mineral water, chew a piece of crackers, and then take a sip of water again.
2. Please write down the code of the sample in a designated spot, e.g. **Sample code:** 523
3. After that unwrap sample, smell burger, mark X in the box on the appropriate liking scale.
4. Next, look at a sample and assess appearance liking.
5. Then taste a sample and evaluate the other sensory traits.
6. Finally, think about overall sensory impression of the sample you have just tried and answer for remaining questions.
7. Please proceed in the same way with the next samples.
8. At the end of assessment, please check if all fields on the card have been completed.
9. We really appreciate **YOUR** individual opinion, so please do not talk to anyone else during the research session.

Sample code: ______

Aroma-liking:
dislike ○ ○ ○ ○ ○ ○ ○ ○ ○ ○ like very much

Appearance-liking:
dislike ○ ○ ○ ○ ○ ○ ○ ○ ○ ○ like very much

Flavour-liking:
dislike ○ ○ ○ ○ ○ ○ ○ ○ ○ ○ like very much

Texture-liking:
dislike ○ ○ ○ ○ ○ ○ ○ ○ ○ ○ like very much

Overall-liking:
dislike ○ ○ ○ ○ ○ ○ ○ ○ ○ ○ like very much

Saltiness:
not salty ○ ○ ○ ○ ○ ○ ○ ○ ○ ○ very salty

How likely are you to buy the burger
highly unlikely ○ ○ ○ ○ ○ ○ ○ highly likely

Please indicate which you think apply to the burger sample you have just tried by placing a cross like this [X] in the box next to it. **There are no right or wrong answers. Please tick as many or as few as you wish.**

	enthusiastic
	happy
	good natured
	free
	joyful
	interested
	understanding
	pleasant
	good

	adventurous
	secure
	active
	satisfied
	loving
	warm
	calm
	aggressive

	nostalgic
	wild
	tame
	mild
	guilty
	worried
	bored
	disgusted

If you have any additional comments regarding this sample, feel free to share your opinion

____

Sample code: ______

Aroma-liking:

dislike ○ ○ ○ ○ ○ ○ ○ ○ ○ ○ like very much

Appearance-liking:

dislike ○ ○ ○ ○ ○ ○ ○ ○ ○ ○ like very much

Flavour-liking:

dislike ○ ○ ○ ○ ○ ○ ○ ○ ○ ○ like very much

Texture-liking:

dislike ○ ○ ○ ○ ○ ○ ○ ○ ○ ○ like very much

Overall-liking:

dislike ○ ○ ○ ○ ○ ○ ○ ○ ○ ○ like very much

Saltiness:

not salty ○ ○ ○ ○ ○ ○ ○ ○ ○ ○ very salty

How likely are you to buy the burger

highly unlikely ○ ○ ○ ○ ○ ○ ○ highly likely

Please indicate which you think apply to the burger sample you have just tried by placing a cross like this [X] in the box next to it. **There are no right or wrong answers. Please tick as many or as few as you wish.**

	disgusted
	pleasant
	warm
	free
	worried
	interested
	aggressive
	enthusiastic
	mild

	happy
	adventurous
	calm
	nostalgic
	good
	good natured
	joyful
	satisfied

	wild
	understanding
	bored
	loving
	tame
	guilty
	active
	secure

If you have any additional comments regarding this sample, feel free to share your opinion

____

Sample code: ______

Aroma-liking:
dislike ○ ○ ○ ○ ○ ○ ○ ○ ○ ○ like very much

Appearance-liking:
dislike ○ ○ ○ ○ ○ ○ ○ ○ ○ ○ like very much

Flavour-liking:
dislike ○ ○ ○ ○ ○ ○ ○ ○ ○ ○ like very much

Texture-liking:
dislike ○ ○ ○ ○ ○ ○ ○ ○ ○ ○ like very much

Overall-liking:
dislike ○ ○ ○ ○ ○ ○ ○ ○ ○ ○ like very much

Saltiness:
not salty ○ ○ ○ ○ ○ ○ ○ ○ ○ ○ very salty

How likely are you to buy the burger
highly unlikely ○ ○ ○ ○ ○ ○ ○ highly likely

Please indicate which you think apply to the burger sample you have just tried by placing a cross like this [X] in the box next to it. **There are no right or wrong answers. Please tick as many or as few as you wish.**

	adventurous
	secure
	disgusted
	tame
	loving
	interested
	happy
	guilty
	good

	worried
	wild
	active
	satisfied
	joyful
	mild
	warm
	enthusiastic

	nostalgic
	good natured
	free
	aggressive
	pleasant
	calm
	understanding
	bored

If you have any additional comments regarding this sample, feel free to share your opinion

____

Sample code: ______

Aroma-liking:
dislike ○ ○ ○ ○ ○ ○ ○ ○ ○ ○ like very much

Appearance-liking:
dislike ○ ○ ○ ○ ○ ○ ○ ○ ○ ○ like very much

Flavour-liking:
dislike ○ ○ ○ ○ ○ ○ ○ ○ ○ ○ like very much

Texture-liking:
dislike ○ ○ ○ ○ ○ ○ ○ ○ ○ ○ like very much

Overall-liking:
dislike ○ ○ ○ ○ ○ ○ ○ ○ ○ ○ like very much

Saltiness:
not salty ○ ○ ○ ○ ○ ○ ○ ○ ○ ○ very salty

How likely are you to buy the burger
highly unlikely ○ ○ ○ ○ ○ ○ ○ highly likely

Please indicate which you think apply to the burger sample you have just tried by placing a cross like this [X] in the box next to it. **There are no right or wrong answers. Please tick as many or as few as you wish.**

	bored
	good natured
	secure
	free
	happy
	guilty
	interested
	enthusiastic
	wild

	nostalgic
	warm
	active
	worried
	aggressive
	good
	disgusted
	calm

	adventurous
	satisfied
	tame
	mild
	understanding
	loving
	joyful
	pleasant

If you have any additional comments regarding this sample, feel free to share your opinion

____

Sample code: ______

Aroma-liking:
dislike ○ ○ ○ ○ ○ ○ ○ ○ ○ ○ like very much

Appearance-liking:
dislike ○ ○ ○ ○ ○ ○ ○ ○ ○ ○ like very much

Flavour-liking:
dislike ○ ○ ○ ○ ○ ○ ○ ○ ○ ○ like very much

Texture-liking:
dislike ○ ○ ○ ○ ○ ○ ○ ○ ○ ○ like very much

Overall-liking:
dislike ○ ○ ○ ○ ○ ○ ○ ○ ○ ○ like very much

Saltiness:
not salty ○ ○ ○ ○ ○ ○ ○ ○ ○ ○ very salty

How likely are you to buy the burger
highly unlikely ○ ○ ○ ○ ○ ○ ○ highly likely

Please indicate which you think apply to the burger sample you have just tried by placing a cross like this [X] in the box next to it. **There are no right or wrong answers. Please tick as many or as few as you wish.**

	adventurous
	happy
	tame
	joyful
	disgusted
	interested
	calm
	aggressive
	free

	loving
	secure
	active
	satisfied
	nostalgic
	worried
	bored
	mild

	enthusiastic
	wild
	good natured
	pleasant
	guilty
	understanding
	good
	warm

If you have any additional comments regarding this sample, feel free to share your opinion

____

Sample code: ______

Aroma-liking:
dislike ○ ○ ○ ○ ○ ○ ○ ○ ○ ○ like very much

Appearance-liking:
dislike ○ ○ ○ ○ ○ ○ ○ ○ ○ ○ like very much

Flavour-liking:
dislike ○ ○ ○ ○ ○ ○ ○ ○ ○ ○ like very much

Texture-liking:
dislike ○ ○ ○ ○ ○ ○ ○ ○ ○ ○ like very much

Overall-liking:
dislike ○ ○ ○ ○ ○ ○ ○ ○ ○ ○ like very much

Saltiness:
not salty ○ ○ ○ ○ ○ ○ ○ ○ ○ ○ very salty

How likely are you to buy the burger
highly unlikely ○ ○ ○ ○ ○ ○ ○ highly likely

Please indicate which you think apply to the burger sample you have just tried by placing a cross like this [X] in the box next to it. **There are no right or wrong answers. Please tick as many or as few as you wish.**

	calm
	happy
	bored
	free
	guilty
	mild
	understanding
	nostalgic
	satisfied

	good natured
	disgusted
	joyful
	good
	adventurous
	pleasant
	secure
	enthusiastic

	aggressive
	loving
	active
	tame
	wild
	worried
	interested
	warm

If you have any additional comments regarding this sample, feel free to share your opinion

____

Thank you for participating in the burger testing!
