# Supplementary figures and images for: Incorporation of Sea Spaghetti (Himanthalia elongata) in Low-Salt Beef Patties: Effect on Sensory Profile and Consumer Hedonic and Emotional Response
Source: Foods. 2024 Apr 15;13(8):1197. doi: 10.3390/foods13081197 (PMC11049442; doi:10.3390/foods13081197)

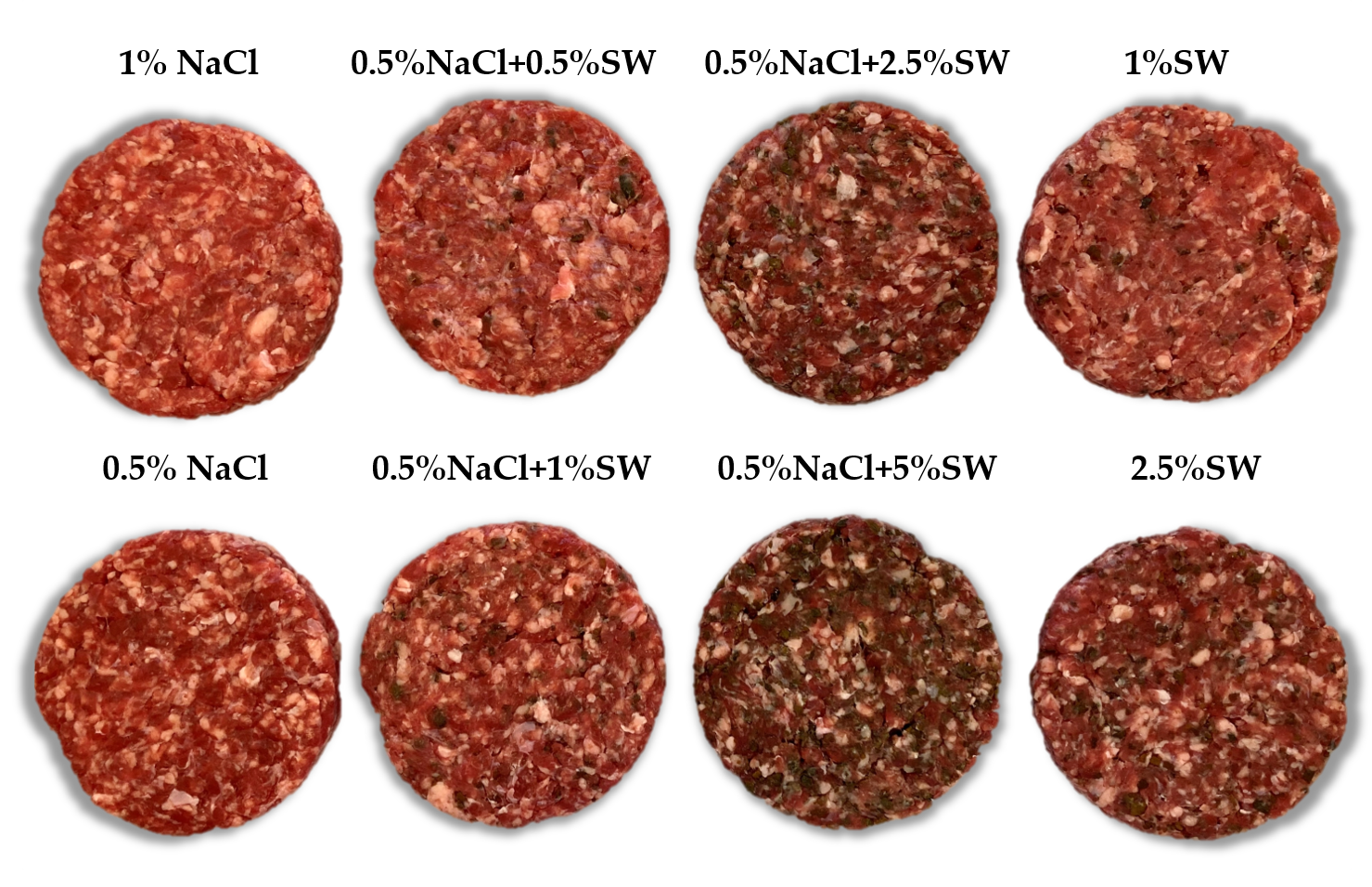

Supplement: Supplementary file 1 [file foods-13-01197-s001.zip › Photo S1. Photograph of raw burgers used in the study.png]
